# Vitamin D in Cardiovascular Medicine: From Molecular Mechanisms to Clinical Translation

**DOI:** 10.3390/nu18030499

**Published:** 2026-02-02

**Authors:** Fahimeh Varzideh, Pasquale Mone, Urna Kansakar, Gaetano Santulli

**Affiliations:** 1 School of Medicine, City University of New York, Manhattan, NY 10031, USA; 2 Casa di Cura “Clinica Montevergine”, 83013 Mercogliano, Avellino, Italy

**Keywords:** cardiology, endocrinology, kidney, metabolism, pharmacology, vitamin D

## Abstract

Vitamin D, a fat-soluble secosteroid traditionally recognized for skeletal health, exerts pleiotropic effects on cardiovascular physiology and disease. Circulating 25-hydroxyvitamin D [25(OH)D], the principal biomarker of vitamin D status, is frequently suboptimal worldwide, particularly in older adults, individuals with darker skin pigmentation, and populations at higher latitudes. Observational studies consistently associate low 25(OH)D concentrations with increased risk of hypertension, atherosclerosis, myocardial infarction, heart failure, arrhythmias, stroke, and cardiovascular mortality. Mechanistic investigations have revealed that vitamin D modulates cardiomyocyte calcium handling, endothelial function, vascular smooth muscle proliferation, inflammation, oxidative stress, and renin–angiotensin–aldosterone system activity, establishing biologically plausible links to cardiovascular outcomes. Despite these associations, large randomized trials of vitamin D supplementation have failed to demonstrate reductions in major cardiovascular events, likely due to heterogeneity in baseline status, dosing regimens, intervention timing, genetic variability, and underlying comorbidities. Vitamin D may function more effectively as a biomarker of cardiovascular risk rather than a universal therapeutic agent, with deficiency reflecting systemic vulnerability rather than acting as a dominant causal factor. Emerging evidence supports precision approaches targeting individuals with severe deficiency, high renin activity, early endothelial dysfunction, or specific genetic profiles, potentially in combination with lifestyle or pharmacologic interventions. Future research should focus on defining optimal dosing strategies, intervention timing, and mechanistic biomarkers to identify subpopulations most likely to benefit, integrating vitamin D therapy into multifaceted cardiovascular prevention frameworks. This systematic review synthesizes molecular, observational, and clinical trial evidence, critically evaluating the current understanding of vitamin D in cardiovascular medicine and highlighting opportunities for targeted, personalized interventions. Vitamin D represents a complex, context-dependent modulator of cardiovascular health, offering both prognostic insight and potential therapeutic value when appropriately applied.

## 1. Introduction

Vitamin D is a fat-soluble secosteroid hormone traditionally recognized for its essential role in calcium and phosphate homeostasis and skeletal maintenance [1]. Historically, its clinical relevance was confined to prevention of rickets and osteomalacia, with deficiency viewed primarily as a bone disorder. Advances in molecular endocrinology and population research have revealed that vitamin D exerts pleiotropic actions in multiple organ systems, including the cardiovascular system. A substantial proportion of the global population exhibits suboptimal serum concentrations of 25-hydroxyvitamin D [25(OH)D], the principal circulating metabolite used to assess vitamin D status, with deficiency particularly prevalent at higher latitudes, in individuals with darker skin pigmentation, and in older adults [2]. These patterns overlap with populations at increased cardiovascular risk, suggesting that vitamin D deficiency may contribute to cardiovascular vulnerability rather than simply coexist with it.

Large observational cohorts demonstrate inverse associations between 25(OH)D concentrations and a wide spectrum of cardiovascular outcomes, including hypertension, coronary artery disease, heart failure, stroke, and both cardiovascular and all-cause mortality [3,4,5,6]. These associations persist after adjustment for age, obesity, physical activity, and socioeconomic status, indicating that vitamin D status may capture biological processes not fully accounted for by conventional risk factors. At the same time, low 25(OH)D concentrations correlate strongly with markers of frailty, chronic inflammation, renal dysfunction, and reduced outdoor activity, raising concerns about residual confounding and reverse causation [7]. Randomized controlled trials (RCTs) of vitamin D supplementation were therefore anticipated to provide definitive answers; yet, most large-scale supplementation trials have failed to demonstrate robust reductions in major cardiovascular events [8,9,10,11,12,13,14]. This disconnect has prompted critical re-examination of trial design, baseline vitamin D status, dosing strategies, achieved serum levels, and the biological plausibility of late intervention. Rather than refuting a role for vitamin D in cardiovascular biology, these findings underscore the complexity of translating endocrine and molecular insights into population-level benefits, highlighting the need for an integrated synthesis of mechanistic data, observational evidence, and clinical trial results.

For this review (registered in PROSPERO with ID: CRD420261294055), we performed a comprehensive literature search to identify studies evaluating the association between vitamin D status, vitamin D supplementation, and cardiovascular outcomes. The electronic databases PubMed/MEDLINE, Embase, Web of Science, and the Cochrane Central Register of Controlled Trials were searched from inception through the most recent search date. The search strategy combined controlled vocabulary and free-text terms related to vitamin D and cardiovascular disease, including “vitamin D,” “25-hydroxyvitamin D,” “cholecalciferol,” “ergocalciferol,” “cardiovascular disease,” “heart failure,” “hypertension,” “coronary artery disease,” “atherosclerosis,” “myocardial infarction,” and “stroke.” Reference lists of relevant reviews and included articles were manually screened to identify additional eligible studies. Eligible studies included randomized controlled trials, prospective and retrospective observational studies, and meta-analyses reporting cardiovascular outcomes or intermediate cardiovascular endpoints in relation to circulating vitamin D levels or vitamin D supplementation in adult populations. Studies were excluded if they focused exclusively on non-cardiovascular outcomes or conditions unrelated to cardiovascular medicine. Two reviewers independently screened titles and abstracts for eligibility, followed by full-text assessment of potentially relevant studies. Discrepancies were resolved by consensus or consultation with a third reviewer. Data extracted included study design, population characteristics, sample size, baseline vitamin D status, intervention type and dose (when applicable), duration of follow-up, cardiovascular outcomes assessed, and key findings. The review was conducted in accordance with the PRISMA guidelines. The methodological quality and risk of bias of randomized controlled trials were assessed using the Cochrane Risk of Bias tool, while observational studies were evaluated using the Newcastle–Ottawa Scale. Given the heterogeneity in study populations, vitamin D definitions, dosing regimens, and cardiovascular endpoints, a qualitative synthesis was primarily undertaken. Where appropriate and methodologically justified, results from randomized trials were summarized separately from observational evidence to facilitate interpretation. Particular attention was paid to sources of heterogeneity, including baseline vitamin D deficiency, comorbid conditions, concomitant therapies, and differences between primary and secondary prevention settings. The overall strength and consistency of the evidence were interpreted in the context of biological plausibility and clinical relevance.

## 2. Vitamin D Biology and Metabolism

**Figure 1:**
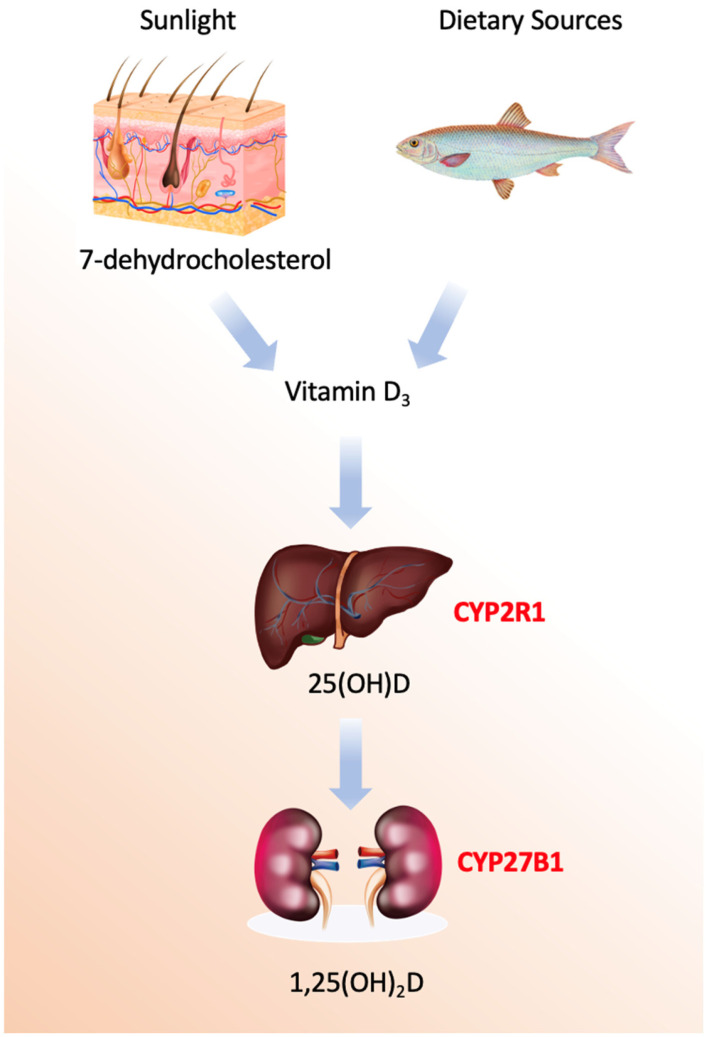
Vitamin D activation pathways.

Vitamin D refers to a group of secosteroids, of which vitamin D3 (cholecalciferol) is synthesized endogenously in the skin following ultraviolet B (UVB)-mediated photoconversion of 7-dehydrocholesterol, while vitamin D2 (ergocalciferol) is derived from plant and fungal sources [1]. Both forms are biologically inert and require sequential hydroxylations to acquire hormonal activity (Figure 1).

The enzymatic machinery governing vitamin D metabolism comprises a sophisticated cascade of cytochrome P450 enzymes with distinct spatial, temporal, and regulatory characteristics [15,16,17,18,19,20,21,22,23,24,25]. The process begins not with an enzyme, but with a photochemical reaction in the skin, where ultraviolet B radiation induces the non-enzymatic conversion of 7-dehydrocholesterol to previtamin D_3_, followed by a thermal isomerization to form vitamin D_3_, or cholecalciferol. This precursor is then transported to the liver bound to vitamin D-binding protein (DBP), a 58 kDa glycoprotein encoded by the *GC* gene on chromosome 4q13.3, which exhibits high-affinity binding specifically for vitamin D metabolites and serves as their systemic carrier.

The first and critical enzymatic activation step occurs primarily in the liver, catalyzed by 25-hydroxylases [19]. The principal enzyme is microsomal CYP2R1, a class II cytochrome P450 located in the endoplasmic reticulum of hepatocytes. This enzyme performs a regioselective hydroxylation at the carbon-25 position of vitamin D_3_, utilizing NADPH-cytochrome P450 reductase and molecular oxygen. Mutations in the *CYP2R1* gene are a known cause of vitamin D-dependent rickets type 1B, underscoring its physiological necessity. A secondary mitochondrial enzyme, CYP27A1, also contributes to this hydroxylation. While CYP27A1 has a broader substrate specificity as a sterol 27-hydroxylase involved in bile acid synthesis, it exhibits lower affinity for vitamin D. Minor contributions may also come from other hepatic P450s, such as the drug-metabolizing enzyme CYP3A4.

The second hydroxylation, which produces the biologically active hormonal form, is the domain of mitochondrial CYP27B1 (1α-hydroxylase). This class I P450 enzyme is predominantly expressed in the proximal tubule cells of the kidney, though it is also found in extra-renal sites like macrophages, keratinocytes, and the placenta. Its catalytic cycle requires an electron transport chain comprising ferredoxin reductase and ferredoxin to deliver electrons from NADPH [20]. The expression and activity of CYP27B1 are tightly regulated; it is potently stimulated by parathyroid hormone (PTH) in response to low blood calcium and is directly inhibited by its own end product, 1,25-dihydroxyvitamin D, forming a critical negative feedback loop. Additional regulation comes from phosphate levels and fibroblast growth factor 23 (FGF23).

Conversely, the catabolic inactivation of vitamin D metabolites is primarily mediated by mitochondrial CYP24A1 (24-hydroxylase). This enzyme initiates a multi-step side-chain oxidation pathway targeting both 25-hydroxyvitamin D and the active 1,25-dihydroxyvitamin D, ultimately leading to the formation of water-soluble calcitroic acid for biliary excretion. CYP24A1 is one of the most strongly induced genes by 1,25-dihydroxyvitamin D itself, via vitamin D response elements in its promoter, creating an auto-catabolic loop that prevents hypervitaminosis D. Its critical role in clearance is highlighted by the disorder idiopathic infantile hypercalcemia, caused by inactivating mutations in *CYP24A1*. Additional catabolic pathways involve hepatic phase II conjugation enzymes, such as UDP-glucuronosyltransferases and sulfotransferases, which facilitate biliary excretion.

Beyond the core renal activation, the discovery of extra-renal CYP27B1 expression has unveiled important paracrine and autocrine functions of vitamin D [21,22]. In immune cells like macrophages, locally produced 1,25-dihydroxyvitamin D acts as an intracrine immunomodulator, a process notably independent of the classic renal feedback controls. This local activation system allows tissues such as skin, colon, and prostate to generate the active hormone for cell-specific purposes like differentiation and apoptosis without affecting systemic calcium homeostasis.

The entire enzymatic network represents a highly evolved endocrine and paracrine system. It integrates signals from calcium, phosphate, PTH, and FGF23 to precisely regulate the production and degradation of 1,25-dihydroxyvitamin D. This balance ensures the maintenance of mineral homeostasis while also supporting the vitamin’s pleiotropic roles in immune modulation, cellular growth, and differentiation. Dysregulation of these enzymes underlies several genetic and acquired diseases, and they remain active targets for therapeutic intervention in conditions ranging from renal failure and hyperparathyroidism to psoriasis and cancer.

**Figure 2:**
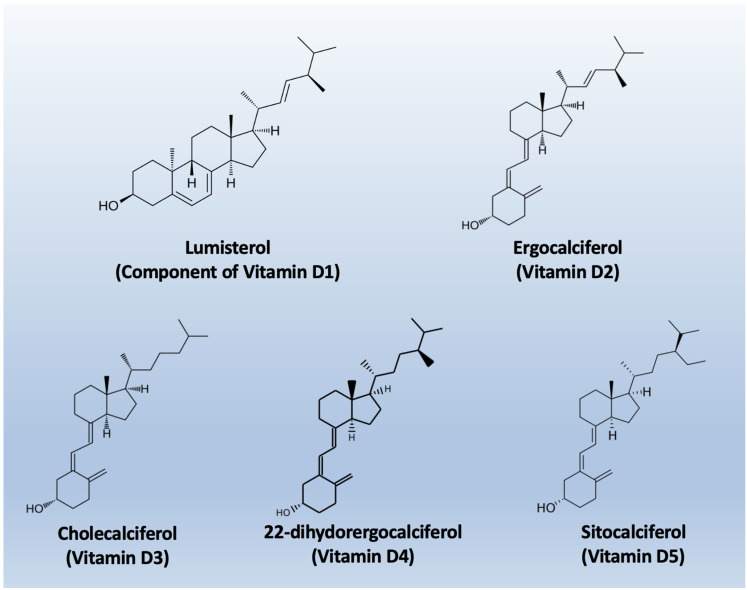
Chemical structures of the main types of Vitamin D.

The nomenclature of vitamin D metabolites and related compounds is reported in Table 1, while the chemical structures are shown in Figure 2.

1,25(OH)_2_D binds primarily to the vitamin D receptor (VDR), a ligand-activated nuclear transcription factor in the steroid hormone receptor superfamily. Upon ligand binding, VDR heterodimerizes with the retinoid X receptor and binds to vitamin D response elements in the promoter regions of target genes, regulating transcriptional programs involved in cell proliferation, differentiation, immune modulation, and extracellular matrix turnover [26,27,28,29,30,31]. VDR is expressed in cardiomyocytes, endothelial cells, vascular smooth muscle cells, and fibroblasts, providing a direct substrate for cardiovascular effects. In addition to genomic effects, rapid non-genomic signaling via membrane-associated VDR modulates intracellular second messengers, allowing acute regulation of cardiovascular function [28,32,33,34,35].

Vitamin D metabolism is tightly regulated by catabolic pathways, primarily through CYP24A1, which hydroxylates both 25(OH)D and 1,25(OH)_2_D, leading to inactivation and excretion [36,37,38,39]. Dysregulation occurs in chronic kidney disease, inflammation, and aging, potentially impairing tissue-specific signaling even when circulating 25(OH)D appears adequate, highlighting limitations in using serum 25(OH)D as a proxy for local cardiovascular activity [40,41,42,43,44]. The main aspects of Vitamin D biology and metabolism are summarized in Table 2.

## 3. Functional Roles of Vitamin D in Cardiovascular Development and Physiology

Vitamin D signaling is crucial in cardiovascular development. Disruption of VDR during embryogenesis alters myocardial structure and vascular patterning, indicating transcriptional regulation of proliferation, differentiation, and extracellular matrix genes essential for cardiac chamber formation and vascular integrity [45]. Epidemiologic studies link maternal deficiency to adverse cardiometabolic outcomes in offspring, supporting the concept of long-lasting cardiovascular effects from early-life vitamin D status [46].

In adults, vitamin D influences myocardial calcium handling, a central determinant of excitation–contraction coupling. Studies in cardiomyocyte show that 1,25(OH)_2_D modulates L-type calcium channels and sarcoplasmic reticulum calcium ATPases, impacting intracellular calcium transients and contractility [47,48,49,50,51,52,53,54,55,56]. Vitamin D also regulates vascular physiology; endothelial cells express VDR and local CYP27B1, enabling autocrine modulation of nitric oxide synthase and oxidative stress pathways, promoting vasodilation and reducing endothelial dysfunction [22,57,58,59,60,61,62,63,64]. In vascular smooth muscle, vitamin D inhibits proliferation and migration through cell cycle regulation and mitogen-activated protein kinase pathways, which may explain associations between deficiency and increased arterial stiffness [65,66,67,68,69,70,71,72,73]. These data suggest that vitamin D maintains cardiovascular structure and function through integrated effects on myocardium, endothelium, and vascular smooth muscle.

## 4. Molecular and Cellular Mechanisms Linking Vitamin D to Cardiovascular Disease

The biological actions of Vitamin D extend far beyond its classical role in calcium-phosphate homeostasis, influencing numerous molecular and cellular processes relevant to cardiovascular health. These processes include modulation of inflammation, oxidative stress, endothelial function, renin–angiotensin–aldosterone system (RAAS) activity, myocardial remodeling, apoptosis, and epigenetic regulation. These mechanisms position vitamin D as a pleiotropic regulator of cardiovascular biology, with deficiency amplifying pathological pathways leading to atherosclerosis, hypertension, myocardial injury, and heart failure. Below, we provide a detailed account of these mechanisms [74,75,76,77,78,79,80,81,82,83,84,85,86].

### 4.1. Immunomodulation and Inflammation

Inflammation is a key driver of atherosclerosis and plaque instability. Vitamin D modulates both innate and adaptive immune responses via the active metabolite 1,25-dihydroxyvitamin D [1,25(OH)_2_D] binding to the vitamin D receptor (VDR), expressed in immune cells, endothelial cells, and vascular smooth muscle cells. 1,25(OH)_2_D suppresses nuclear factor kappa B (NF-κB) signaling, reducing transcription of pro-inflammatory cytokines such as tumor necrosis factor-alpha (TNF-α), interleukin-6 (IL-6), and interleukin-1β (IL-1β). In cultured human endothelial cells, vitamin D attenuates TNF-α-induced NF-κB activation and decreases expression of adhesion molecules like VCAM-1 and E-selectin, reducing leukocyte recruitment and early atherogenic events [87,88,89,90,91,92,93,94,95,96,97,98,99]. Vitamin D also shifts macrophages and dendritic cells toward anti-inflammatory phenotypes, decreasing secretion of MCP-1, IL-6, and IL-8 and reducing THP-1 monocyte migration in vitro, thereby limiting vascular inflammation [100,101,102,103,104,105,106,107,108,109,110,111,112,113,114]. Adaptive immunity is similarly affected, with 1,25(OH)_2_D promoting regulatory T-cell differentiation and reducing Th1/Th17 responses, modulating cytokine networks implicated in plaque destabilization [115,116,117,118,119].

Epidemiological studies support these mechanistic insights, showing that low serum 25(OH)D correlates with elevated high-sensitivity C-reactive protein (hs-CRP) and pro-inflammatory cytokines, linking deficiency to systemic inflammatory burden that contributes to cardiovascular risk [120,121]. This evidence positions vitamin D deficiency as both a marker and modulator of inflammatory cardiovascular processes.

### 4.2. Oxidative Stress and Mitochondrial Function

Oxidative stress and mitochondrial dysfunction are fundamental drivers of endothelial injury and play a central role in the development of atherosclerosis as well as in myocardial ischemia–reperfusion injury. Excessive production of reactive oxygen species (ROS) disrupts mitochondrial integrity, impairs cellular energy metabolism, and promotes inflammatory and apoptotic signaling within both vascular and myocardial tissues. Accumulating evidence indicates that vitamin D exerts important protective effects in this context by enhancing mitochondrial function and strengthening endogenous antioxidant defenses [122,123,124,125,126,127,128,129,130,131,132,133]. Experimental studies in cardiomyocytes demonstrate that deficiency of circulating 25-hydroxyvitamin D [25(OH)D] leads to impaired oxidative phosphorylation, reduced mitochondrial efficiency, and increased ROS generation, thereby creating a cellular environment that predisposes to oxidative injury and cardiomyocyte dysfunction [134].

At the molecular level, the active vitamin D metabolite 1,25-dihydroxyvitamin D [1,25(OH)_2_D] modulates redox homeostasis through transcriptional regulation of key antioxidant systems. Specifically, 1,25(OH)_2_D upregulates the expression and activity of major antioxidant enzymes, including superoxide dismutase (SOD), glutathione peroxidase, and catalase, resulting in enhanced detoxification of superoxide anions and hydrogen peroxide and attenuation of oxidative damage in both myocardial and vascular tissues [135,136]. A critical mechanism underlying these effects involves activation of the nuclear factor erythroid 2-related factor 2 (Nrf2) signaling pathway. Vitamin D–dependent activation of Nrf2 promotes nuclear translocation and binding to antioxidant response elements, thereby increasing transcription of a broad array of cytoprotective antioxidant genes and reducing ROS-mediated endothelial apoptosis [137].

In vascular smooth muscle cells, vitamin D signaling further limits oxidative stress by suppressing NADPH oxidase activity, a major enzymatic source of vascular superoxide. This reduction in superoxide generation preserves nitric oxide bioavailability, improves endothelial-dependent vasodilation, and contributes to the maintenance of vascular homeostasis [138]. Collectively, these findings position vitamin D as an important modulator of mitochondrial integrity and redox balance in cardiovascular tissues, with direct implications for endothelial function and cardioprotection.

### 4.3. Endothelial Function and Vascular Homeostasis

Vitamin D exerts direct and multifaceted protective effects on the vascular endothelium, a critical regulator of vascular tone, permeability, inflammation, and thrombosis [22]. Activation of the vitamin D receptor (VDR) in endothelial cells promotes nitric oxide (NO) bioavailability by enhancing endothelial nitric oxide synthase (eNOS) phosphorylation and enzymatic activity. These effects are mediated through activation of the phosphoinositide 3-kinase (PI3K)/Akt and mitogen-activated protein kinase (MAPK) signaling pathways, leading to increased NO production and improved endothelium-dependent vasodilation. Enhanced NO signaling also suppresses endothelial activation, reducing leukocyte adhesion and transendothelial migration, key early steps in vascular inflammation and atherogenesis [58,59,66,139].

Beyond its effects on NO signaling, vitamin D plays an important role in maintaining endothelial cell viability and functional integrity under adverse metabolic conditions [62,63,140,141,142,143,144,145,146]. Experimental evidence indicates that vitamin D signaling inhibits endothelial senescence and apoptosis induced by hyperglycemia and oxidative stress, conditions commonly observed in diabetes and cardiometabolic disease [60,141,142,147,148,149,150]. By limiting oxidative damage, mitochondrial dysfunction, and pro-apoptotic signaling, vitamin D preserves endothelial barrier function and vascular homeostasis. Collectively, these actions position vitamin D as a critical regulator of endothelial health, linking vitamin D deficiency to endothelial dysfunction and increased cardiovascular risk.

### 4.4. Regulation of the Renin–Angiotensin–Aldosterone System

Vitamin D functions as a critical negative regulator of the renin–angiotensin–aldosterone system (RAAS), a central hormonal pathway governing blood pressure, vascular tone, sodium balance, and cardiac remodeling. Disruption of vitamin D signaling has profound consequences for RAAS activation and cardiovascular homeostasis [151,152,153,154,155,156,157,158,159,160,161,162,163,164,165,166,167,168,169]. Experimental studies in vitamin D receptor (VDR)–null mice demonstrate marked upregulation of renin expression, leading to elevated angiotensin II levels, hypertension, left ventricular hypertrophy, and increased cardiovascular mortality, underscoring the essential role of VDR signaling in suppressing pathological RAAS activity [170].

At the molecular level, the active vitamin D metabolite 1,25-dihydroxyvitamin D [1,25(OH)_2_D] directly inhibits renin gene transcription. This effect is mediated through VDR binding to regulatory elements within the renin gene promoter, resulting in transcriptional repression independent of sodium balance or angiotensin II feedback mechanisms [171]. These findings establish vitamin D as an endocrine modulator of RAAS at the level of gene regulation.

Observational and clinical studies in humans further support this regulatory axis, demonstrating inverse associations between circulating 25-hydroxyvitamin D [25(OH)D] concentrations and both plasma renin activity and blood pressure. These relationships are particularly pronounced in individuals with vitamin D deficiency, suggesting that insufficient vitamin D status may contribute to inappropriate RAAS activation and increased cardiovascular risk [172]. In addition to suppressing renin, vitamin D influences downstream RAAS components, including modulation of angiotensin-converting enzyme 2 (ACE2) expression. ACE2 counterbalances classical RAAS signaling by promoting formation of vasodilatory and anti-fibrotic angiotensin peptides. Vitamin D–mediated upregulation of ACE2 therefore represents an additional cardioprotective mechanism, with potential implications for vascular function, myocardial remodeling, and inflammation [173,174]. Together, these data highlight vitamin D as a key regulator of RAAS homeostasis and provide mechanistic insight into its role in cardiovascular protection.

### 4.5. Myocardial Remodeling and Fibrosis

Vitamin D plays an important role in limiting maladaptive myocardial remodeling and fibrotic remodeling, processes that underlie the progression of heart failure and adverse cardiovascular outcomes. Experimental studies demonstrate that vitamin D signaling attenuates pathological cardiac hypertrophy and interstitial fibrosis by inhibiting transforming growth factor–beta (TGF-β) signaling, a central profibrotic pathway driving fibroblast activation and myofibroblast differentiation. Through suppression of TGF-β–dependent transcriptional programs, vitamin D reduces cardiac fibroblast proliferation and collagen synthesis, leading to decreased extracellular matrix accumulation and improved ventricular compliance in models of pressure overload and myocardial infarction (MI). In addition to limiting fibroblast activation, vitamin D regulates extracellular matrix turnover by modulating the balance between matrix metalloproteinases (MMPs) and their endogenous tissue inhibitors. By restoring appropriate MMP activity, vitamin D prevents excessive collagen deposition, limits ventricular stiffening, and preserves myocardial architecture during chronic cardiac stress. These effects contribute to maintenance of normal ventricular geometry and diastolic function [175,176,177,178,179].

Vitamin D also exerts direct cytoprotective effects within cardiomyocytes. The active metabolite 1,25-dihydroxyvitamin D [1,25(OH)_2_D] reduces cardiomyocyte apoptosis by modulating members of the Bcl-2 family of proteins, shifting the balance toward anti-apoptotic signaling and enhancing cell survival. Preservation of cardiomyocyte viability helps maintain myocardial mass and contractile performance, particularly under conditions of hemodynamic overload or ischemic injury.

Collectively, these mechanisms are highly relevant to heart failure pathophysiology. Clinical and epidemiologic studies consistently associate vitamin D deficiency with adverse patterns of cardiac remodeling, including increased ventricular dilation, reduced ejection fraction, and worse clinical outcomes, underscoring the potential role of vitamin D signaling in modifying disease progression [175,179,180].

### 4.6. Epigenetic Modulation and Gene Expression

Growing evidence indicates that vitamin D regulates cardiovascular biology not only through classical transcriptional mechanisms but also via epigenetic modulation of gene expression. Activation of the vitamin D receptor (VDR) leads to its binding at specific promoter and enhancer regions across the genome, where it interacts with chromatin-modifying complexes to influence histone acetylation and methylation states [181,182,183,184,185,186]. These changes alter chromatin accessibility and transcriptional activity of genes involved in inflammation, oxidative stress responses, cellular proliferation, and fibrotic remodeling, thereby shaping cardiovascular phenotypes in a context-dependent manner [187,188].

Vitamin D signaling has also been shown to influence DNA methylation patterns at key cardiometabolic loci, contributing to sustained alterations in gene expression that may persist beyond acute exposure. Such epigenetic reprogramming provides a mechanistic framework for understanding how vitamin D status during critical windows may exert long-term effects on cardiovascular risk and disease susceptibility [189,190,191,192,193,194,195]. Interindividual variability in these epigenetic responses may partially explain heterogeneous clinical responses to vitamin D supplementation observed in population studies.

In addition, vitamin D modulates the expression and activity of non-coding RNAs, particularly microRNAs, which serve as fine-tuners of post-transcriptional gene regulation [196,197,198,199,200,201,202,203,204]. Vitamin D–dependent regulation of specific microRNAs has been implicated in controlling endothelial cell proliferation and survival, vascular smooth muscle cell migration and phenotypic switching, and immune cell polarization toward less pro-inflammatory states. Through these effects, vitamin D integrates genomic and non-genomic signaling pathways to coordinate vascular repair, limit pathological remodeling, and maintain vascular homeostasis under physiological and pathological conditions [205,206,207,208,209,210,211,212,213,214,215,216,217,218,219,220].

These epigenetic mechanisms provide a unifying explanation for the broad and durable cardiovascular effects of vitamin D and highlight its potential role in modulating long-term cardiovascular risk through stable, yet reversible, changes in gene regulation.

### 4.7. Integration of Molecular Pathways

The mechanisms described above converge on early, potentially reversible stages of cardiovascular disease. Vitamin D deficiency amplifies inflammatory signaling, oxidative stress, maladaptive RAAS activation, endothelial dysfunction, and myocardial fibrosis. Conversely, adequate vitamin D status supports anti-inflammatory phenotypes, antioxidant defenses, NO-mediated vasodilation, RAAS suppression, and preservation of myocardial structure. The timing of intervention, baseline deficiency, tissue-specific VDR expression, and genetic polymorphisms all influence the degree to which these molecular actions translate into measurable clinical benefit. These mechanistic insights help reconcile why observational studies show robust associations between low 25(OH)D and cardiovascular risk, whereas randomized supplementation trials in broadly unselected populations often yield neutral results [9].

So, Vitamin D functions as a pleiotropic regulator of cardiovascular biology, modulating immune responses, oxidative stress, endothelial function, neurohormonal signaling, myocardial remodeling, and epigenetic control of gene expression. Deficiency removes these protective layers, creating a permissive environment for vascular inflammation, atherosclerosis, hypertension, and heart failure [221,222,223,224,225,226,227,228]. These molecular and cellular insights underscore the importance of early detection of deficiency and may inform targeted supplementation strategies in at-risk populations, while highlighting the complex interplay of genetic, epigenetic, and environmental factors that determine individual cardiovascular responses [103,229].

## 5. Vitamin D and Specific Cardiovascular Conditions

The relationship between vitamin D and hypertension has been extensively studied. Cross-sectional and prospective cohort studies demonstrate an inverse association between circulating 25-hydroxyvitamin D concentrations and both systolic and diastolic blood pressure, as well as incident hypertension [230,231,232,233,234,235,236,237,238,239,240,241,242]. Mechanistic studies indicate that vitamin D modulates renin transcription, vascular smooth muscle tone, and endothelial nitric oxide production, providing a biological rationale for these epidemiological observations [243]. However, the magnitude of effect observed in observational studies frequently exceeds that reported in randomized trials, suggesting that deficiency may interact with other cardiovascular risk factors to influence blood pressure.

Atherosclerotic cardiovascular disease has also been associated with vitamin D status. Prospective cohort studies link low serum 25-hydroxyvitamin D levels to higher incidence of MI and coronary mortality [244]. At the cellular level, vitamin D influences endothelial function, macrophage lipid handling, and vascular inflammation, processes central to atherogenesis. Clinical trials assessing surrogate markers of atherosclerosis, such as carotid intima–media thickness and coronary artery calcification, have largely yielded neutral results following vitamin D supplementation [245]. These findings suggest that intervention timing is critical and that supplementation may be more effective before the establishment of irreversible structural vascular disease.

Heart failure represents another context in which vitamin D biology is particularly relevant. Observational studies indicate high prevalence of deficiency in heart failure populations, with lower 25(OH)D levels correlating with worse functional capacity, higher natriuretic peptide concentrations, and increased mortality [246,247,248,249,250,251,252,253,254,255]. Mechanistic studies demonstrate that deficiency exacerbates myocardial remodeling, impairs calcium handling, and promotes secondary hyperparathyroidism, which itself may worsen outcomes; interventional studies show mixed results; some report improvements in inflammatory markers and left ventricular structure, while others fail to demonstrate clinical endpoint benefits [256,257]. These discrepancies underscore the importance of early intervention and the multifactorial nature of heart failure pathophysiology.

Cardiac arrhythmias, including atrial fibrillation and sudden cardiac death, have been associated with low vitamin D status. Vitamin D modulates cardiomyocyte electrophysiology by regulating calcium flux and ion channel expression, providing a mechanistic rationale for arrhythmia risk modulation. Observational studies suggest that deficiency increases incidence of postoperative atrial fibrillation and arrhythmias in critically ill patients [258]. However, causal inference is limited, and randomized trials specifically targeting arrhythmic endpoints are lacking.

Vitamin D deficiency has also been linked to cerebrovascular disease. Prospective analyses associate low 25-hydroxyvitamin D with increased risk of ischemic stroke and worse outcomes following cerebrovascular events [259]. Mechanisms include endothelial dysfunction, prothrombotic shifts, and increased arterial stiffness. Supplementation trials, however, have not consistently demonstrated reductions in stroke incidence, suggesting that deficiency may act as a risk marker rather than a direct therapeutic target.

## 6. Vitamin D Deficiency as a Cardiovascular Risk Marker

Vitamin D deficiency frequently co-occurs with established cardiovascular risk factors such as obesity, physical inactivity, chronic kidney disease, and systemic inflammation, creating a complex web of interconnected exposures that complicates the attribution of causality in cardiovascular outcomes [260,261].

Obesity is a major determinant of both vitamin D status and cardiovascular risk. Adipose tissue acts as a reservoir for fat-soluble vitamins, including vitamin D, sequestering it and reducing its bioavailability in circulation. Consequently, individuals with obesity often exhibit lower serum 25-hydroxyvitamin D levels despite adequate intake or sun exposure. This deficiency is not merely a biochemical finding; it has important clinical implications. Low vitamin D is associated with endothelial dysfunction, increased inflammation, insulin resistance, and dysregulation of the renin–angiotensin–aldosterone system, all of which are recognized contributors to cardiovascular disease. Moreover, obesity independently elevates cardiovascular risk through mechanisms such as hypertension, dyslipidemia, chronic low-grade inflammation, and structural cardiac changes. The combination of vitamin D deficiency and obesity may therefore synergistically amplify cardiovascular risk, highlighting the need for integrated strategies targeting weight reduction, metabolic health, and optimal vitamin D status [262,263,264,265,266,267,268,269,270,271,272,273,274,275,276,277,278,279].

In large prospective cohort studies, low circulating 25-hydroxyvitamin D concentrations have been consistently associated with increased relative risks of cardiovascular disease (CVD) incidence and mortality, with one meta-analysis finding that individuals in the lowest categories of 25(OH)D had approximately a 44% higher risk of combined CVD incidence and mortality compared with those in the highest categories (relative risk [RR] = 1.44, 95% CI: 1.24–1.69), and a 54% higher rate of CVD mortality specifically (RR = 1.54, 95% CI: 1.29–1.84) [5,260,280,281]. These associations are generally robust to adjustment for multiple traditional risk factors including age, sex, smoking, and lipid levels, but residual confounding remains a persistent concern because many of the factors that reduce vitamin D levels—such as lower physical activity or adiposity—also independently increase cardiovascular risk.

Reverse causation is another plausible contributor, particularly in advanced disease states where individuals with chronic illness may have reduced mobility, spend less time outdoors, and thereby have lower ultraviolet B exposure and reduced cutaneous synthesis of vitamin D. In longitudinal cohorts of individuals with prehypertension, severe serum 25(OH)D deficiency was independently associated with higher all-cause and cardiovascular mortality even after adjusting for multiple covariates, suggesting that deficiency correlates with adverse outcomes even in subclinical disease states [282]. Likewise, in U.S. National Health and Nutrition Examination Survey analyses among individuals with chronic kidney disease, lower 25(OH)D was linked to both all-cause and cardiovascular mortality in adjusted models, with a non-linear inverse association that persisted across quartiles of vitamin D status [6].

The role of inflammation may help explain how low vitamin D acts as a marker rather than a causal agent. Chronic low-grade inflammation, marked by elevated high-sensitivity C-reactive protein (hs-CRP), is itself a strong predictor of cardiovascular events, and combined vitamin D deficiency plus high hs-CRP dramatically increases both all-cause and CVD-specific mortality more than either exposure alone (e.g., a 130% increase in CVD mortality when both are present), highlighting how 25(OH)D integrates with systemic inflammatory burden [283]. An inverse correlation between 25(OH)D and pro-inflammatory cytokines such as interleukin-6 and tumor necrosis factor-α (which are implicated in atherogenesis and plaque instability) has been proposed, further embedding low vitamin D within broader inflammatory risk profiles [284].

Mendelian randomization (MR) studies, which use genetic variants as proxies for lifelong differences in exposure to reduce confounding and reverse causation, have provided mixed insights into causality. Traditional MR analyses across large cohorts including UK Biobank and other population samples have generally found no significant causal association between genetically predicted 25(OH)D levels and coronary heart disease, stroke, or mortality, suggesting that observational associations likely reflect confounding or correlated exposures rather than direct cause–effect relationships [285,286]. However, more recent non-linear MR designs indicate that the relationship may be L-shaped, with the steepest decrease in cardiovascular risk occurring as 25(OH)D concentrations rise from severely deficient to moderately sufficient levels, and risk plateauing beyond ~50 nmol/L, implying that causal effects may be concentrated at deficiency extremes [287,288]. These more nuanced MR approaches suggest that while average lifelong differences in 25(OH)D may not causally impact CVD risk across the full range, correcting severe deficiency could plausibly reduce events.

Seasonal variation further illustrates the complexity of interpreting vitamin D status as a risk marker for CVD. Numerous epidemiological studies document seasonal fluctuations in serum 25(OH)D—with higher concentrations in summer and lower in winter—paralleling well-replicated seasonal patterns in cardiovascular events such as MI and stroke, which peak in colder months and decline in warmer months; however, other co-varying factors such as ambient temperature, infectious disease prevalence, and physical activity also shift seasonally and likely contribute to observed CVD risk patterns [289,290,291]. The mere co-occurrence of seasonal vitamin D changes with seasonal cardiovascular patterns does not prove causality but underscores the biomarker’s sensitivity to environmental exposures that also influence cardiovascular systems.

Additionally, large prospective cohort studies and meta-analyses have documented that associations between 25(OH)D and CVD outcomes often diminish or become non-significant after adjustment for socioeconomic indicators, metabolic risk profiles, and behavioral determinants of health, reaffirming that low vitamin D is an integrative marker of poorer health status rather than a stand-alone causal driver in many contexts [292,293]. In this vein, low 25(OH)D is tightly linked with metabolic syndrome components, dyslipidemia, and insulin resistance, which are themselves causal risk factors for cardiovascular disease, making it difficult to discern whether vitamin D deficiency independently predicts risk or merely tags a cluster of maladaptive metabolic and lifestyle factors [294,295,296,297,298,299,300,301,302,303,304,305,306,307,308,309,310].

Despite the causal ambiguity, 25(OH)D remains a robust prognostic biomarker in many cardiovascular risk models, because its levels reflect a confluence of lifestyle, nutritional, inflammatory, and comorbid disease processes. Lower 25(OH)D often identifies individuals who have multiple risk enhancers—such as high adiposity, limited outdoor activity, chronic inflammation, and comorbid chronic disease—who may be at increased risk of adverse cardiovascular outcomes. This makes 25(OH)D valuable in risk stratification even if it does not itself causally drive pathology. The concept that vitamin D deficiency could act as a surrogate marker of cumulative cardiometabolic stress rather than a primary etiological agent aligns with the pattern of observational associations and the mostly null results of randomized supplementation trials in broad populations, where correcting deficiency alone did not meaningfully alter clinical endpoints.

## 7. Clinical Trials Testing Vitamin D Supplementation in Cardiovascular Disease

Randomized controlled trials (RCTs) represent the highest level of evidence for establishing causal effects of interventions. In the context of vitamin D supplementation and cardiovascular disease (CVD), a large body of RCT data has emerged over the past decade, yet the results have largely been neutral for major cardiovascular endpoints. Understanding these trials’ designs, populations, dosing regimens, and outcomes is essential for interpreting their implications and guiding future research.

### 7.1. Early Trials and Surrogate Outcomes

In the early stages of interest in vitamin D for CVD prevention, small RCTs tested surrogate cardiovascular risk markers or intermediate physiological endpoints. Many were conducted in high-risk or patient populations and were not powered for hard endpoints.

For example, a prospective, randomized, placebo-controlled trial of postmenopausal women with serum 25-hydroxyvitamin D [25(OH)D] levels between 10 and 60 ng/mL randomized participants to vitamin D3 2500 IU/day or placebo for four months to evaluate changes in biochemical and vascular risk markers; however, the short duration and small sample limited its ability to detect clinical events. While such small RCTs demonstrated feasibility and safety, they did not provide definitive evidence that vitamin D alters cardiovascular risk factors in a clinically meaningful way, particularly since changes in individual biomarkers did not translate into reduced disease incidence.

These early trials informed the design of larger, more definitive RCTs with cardiovascular endpoints, including primary prevention in largely healthy populations and secondary analyses in disease-specific cohorts.

### 7.2. Primary Prevention in Unselected Populations

The most influential vitamin D supplementation trials for cardiovascular outcomes have been large primary prevention studies in generally healthy adult populations. These trials tested whether routine vitamin D supplementation reduces the incidence of major adverse cardiovascular events (MACE), such as MI, stroke, or cardiovascular mortality.

#### 7.2.1. The VITamin D and OmegA-3 Trial (VITAL)

The *VITamin D and OmegA-3 TriaL (VITAL)* is the largest RCT to date to investigate vitamin D3 supplementation and cardiovascular outcomes in a primary prevention setting. In a 2 × 2 factorial design, 25,871 U.S. adults without prior CVD or cancer were randomized to receive vitamin D3 2000 IU daily or placebo (as well as omega-3 fatty acids or placebo) with a median follow-up of 5.3 years [9]. The primary composite endpoint was major CVD events (MI, stroke, or cardiovascular death). Vitamin D supplementation did not significantly reduce the risk of the primary cardiovascular composite outcome (hazard ratio [HR] 0.97; 95% confidence interval [CI], 0.85–1.10; *p* = 0.69) compared with placebo [9]. Secondary endpoints, including all-cause mortality, were likewise not significantly different between groups, and subgroup analyses by sex, baseline 25(OH)D level, and body mass index did not reveal consistent benefit. Notably, VITAL’s cohort had a relatively high baseline vitamin D status, with mean serum 25(OH)D concentrations above deficiency thresholds in most participants, raising questions about whether supplementation could benefit truly deficient individuals [9].

In addition, an echocardiographic substudy within the VITAL examined cardiac structure and function after two years of intervention and found no significant differences in left ventricular (LV) mass or systolic and diastolic function between vitamin D and placebo arms, further supporting the null effect on structural cardiovascular endpoints [10].

#### 7.2.2. Vitamin D Assessment Study (ViDA)

The *Vitamin D Assessment Study (ViDA)* was a randomized clinical trial conducted in New Zealand that tested monthly high-dose vitamin D3 (initial loading dose of 200,000 IU followed by 100,000 IU monthly) versus placebo in 5108 adults over a median of 3.3 years [11]. The mean baseline 25(OH)D was approximately 26.5 ng/mL, with about 25% of participants classified as deficient.

The primary outcome was incident CVD (including MI, angina, heart failure, stroke, and cardiovascular death). ViDA found no significant reduction in cardiovascular events in the vitamin D group compared with placebo (adjusted HR 1.02; 95% CI: 0.87–1.20). Subgroup analyses among participants with baseline 25(OH)D deficiency also showed no statistically significant benefit, although the confidence intervals were wide, reflecting limited power for subgroup detection [11].

#### 7.2.3. D-Health Trial

The *D-Health Trial* in Australia enrolled over 21,000 adults aged 60–84 years and randomized them to monthly vitamin D3 60,000 IU or placebo for up to five years [14]. The primary prespecified outcomes included major cardiovascular events, defined as MI, stroke, or coronary revascularization. The trial reported a relative hazard of 0.91 (95% CI: 0.81–1.01) for major cardiovascular events with vitamin D supplementation compared with placebo, suggesting a non-statistically significant trend toward lower risk [14].

When secondary endpoints were analyzed, the hazard ratio for MI specifically was 0.81 (95% CI: 0.67–0.98), indicating a potential reduction in MI incidence, though stroke incidence was unchanged and the trial overall did not meet statistical thresholds for primary endpoint reduction [14]. These nuanced findings hint at possible differential effects on specific cardiovascular outcomes, yet the absolute risk reduction was small and consistent with a null effect overall.

#### 7.2.4. Finnish Vitamin D Trial (FIND)

The *Finnish Vitamin D Trial (FIND)* evaluated daily vitamin D3 supplementation at 1600 IU/day or 3200 IU/day versus placebo in older Finnish adults over five years, with the primary outcomes of major CVD events and invasive cancer [13]. Baseline cardiovascular health was generally good, and mean 25(OH)D levels were near sufficiency due to fortification policies in Finland. The trial found no significant reduction in the incidence of major CVD events in either vitamin D intervention arm compared with placebo (hazard ratios ~0.97 and ~0.84 for the two doses, respectively, with wide confidence intervals) [13]. These findings reinforce the general null pattern observed in primary prevention RCTs when baseline status is sufficient or mildly insufficient.

### 7.3. Secondary Prevention and Disease-Specific Trials

While most large primary prevention trials have reported neutral findings, some RCTs have focused on individuals with existing cardiovascular or related conditions to evaluate whether vitamin D supplementation might modify disease progression or severity [311].

#### Heart Failure Trials (EVITA)

The *Effect of Vitamin D on All-Cause Mortality in Heart Failure (EVITA)* trial randomized 400 patients with advanced heart failure and baseline 25(OH)D < 75 nmol/L to vitamin D3 4000 IU daily or placebo for three years [12]. Despite significant increases in serum 25(OH)D in the treatment arm, all-cause mortality did not differ significantly between groups (HR 1.09; 95% CI: 0.69–1.71; *p* = 0.73) [12]. This trial was not designed primarily to assess cardiovascular events but nonetheless failed to demonstrate a survival or major morbidity advantage with supplementation in a high-risk clinical cohort.

Other smaller heart failure trials have examined functional outcomes, biomarkers of inflammation, or left ventricular remodeling. While some have reported modest improvements in surrogate markers such as inflammatory cytokines or muscle strength, these changes have not translated into consistent reductions in heart failure hospitalizations or mortality, emphasizing the need for larger, disease-specific RCTs if benefits are to be demonstrated.

The main trials evaluating the effects of Vitamin D Supplementation on cardiovascular outcomes are reported in Table 3.

### 7.4. Meta-Analyses of Randomized Trials

Given the predominantly neutral results of individual RCTs, several meta-analyses have pooled data to assess overall effects of vitamin D supplementation on cardiovascular outcomes (Table 4); most meta-analyses demonstrate neutral effects on cardiovascular morbidity and mortality despite modest signals in all-cause mortality in broader aggregations of RCTs.

A comprehensive meta-analysis involving 21 RCTs with more than 83,000 participants found that vitamin D supplementation was not associated with reduced risk of MACE, MI, stroke, cardiovascular mortality, or all-cause mortality compared with placebo (risk ratio [RR] ≈ 1.00 for MACE; 95% CI: 0.95–1.06) [315]. The results were consistent across subgroup analyses for sex, baseline 25(OH)D levels, dosing regimen (daily vs. bolus), and co-administration with calcium [315].

Another recent systematic review and meta-analysis of 80 RCTs found that while vitamin D supplementation was associated with a modest reduction in all-cause mortality, it did not significantly reduce specific cardiovascular morbidity or mortality outcomes, including MI, stroke, or heart failure [313]. This pattern suggests that any observed survival benefits may not be driven primarily by cardiovascular disease reduction and may reflect effects on other health domains.

### 7.5. Interpretation and Sources of Heterogeneity

The totality of RCT evidence consistently indicates that vitamin D supplementation does not meaningfully reduce cardiovascular events in broad, unselected adult populations, and several factors help explain the apparent disconnect between these null findings and the positive associations observed in epidemiological studies. One key consideration is baseline vitamin D status: many large RCTs enrolled participants with mean serum 25-hydroxyvitamin D [25(OH)D] concentrations above levels considered deficient, leaving limited room for supplementation to provide additional benefit, as exemplified by trials such as VITAL and FIND, where raising already sufficient or mildly insufficient vitamin D levels did not confer measurable cardiovascular protection [9,13]. Dosing strategies further contribute to heterogeneity, as trials employed a variety of regimens including daily, weekly, and large monthly bolus doses; the pharmacokinetics and physiological effects of bolus administration may differ from daily dosing, potentially affecting receptor activation and downstream cardiovascular pathways, although meta-analyses have generally not found consistent differences in outcomes based on dosing frequency [316]. Intervention timing is another important factor, given that cardiovascular disease develops over decades; supplementation in middle-aged or older adults may be insufficient to reverse established atherosclerosis, endothelial dysfunction, or myocardial remodeling. Outcome power also contributes to variability, as many trials—including VITAL, ViDA, and D-Health—while large, considered cardiovascular events as secondary outcomes or were underpowered to detect differences in individual endpoints such as heart failure hospitalization or sudden cardiac death. Genetic and biological heterogeneity may modulate responses to supplementation, with variants in the vitamin D receptor, vitamin D–binding protein, and enzymes such as CYP27B1 influencing individual outcomes, yet RCTs have not systematically stratified participants based on these genetic differences. Finally, the interplay with other interventions may mask potential effects of vitamin D, as participants in primary prevention trials were often receiving modern preventive therapies, including statins and antihypertensives, which could reduce the incremental cardiovascular benefit attributable to vitamin D supplementation. These factors underscore the complexity of interpreting RCT findings and highlight that while vitamin D supplementation reliably raises serum 25(OH)D levels, translating this biochemical correction into meaningful cardiovascular risk reduction remains challenging in unselected populations.

The most robust evidence to date indicates that vitamin D supplementation, when administered broadly in primary prevention settings, does not significantly reduce major cardiovascular events or mortality. Subgroup hints of benefit (e.g., MI reduction in D-Health) are intriguing but have not been replicated consistently. The RCT literature underscores the need for future precision trials that focus on individuals with profound deficiency, earlier intervention, and mechanistically defined risk profiles to determine whether targeted supplementation can modify specific cardiovascular outcomes.

Several factors may explain the disconnect between observational associations and neutral trial results. Baseline vitamin D status is critical; observational studies demonstrate graded risk increases at lower 25-hydroxyvitamin D concentrations, whereas trials often enroll participants with levels above deficiency thresholds. Dosing strategy is another factor: intermittent high-dose regimens used in some trials produce large serum fluctuations that may alter vitamin D receptor activation and downstream gene expression compared with physiologic daily exposure [317].

Timing of intervention is also relevant. Cardiovascular disease develops over decades, and supplementation in later stages may be insufficient to reverse established structural or functional changes. Genetic and epigenetic heterogeneity further modulates responsiveness; polymorphisms in VDR, CYP27B1, and vitamin D–binding protein influence circulating levels, tissue availability, and receptor-mediated effects [18]. Finally, vitamin D likely functions as a permissive factor, supporting optimal cardiovascular regulation, rather than as a dominant causal agent. Deficiency may exacerbate underlying pathology, but supplementation alone cannot overcome strong drivers such as obesity, diabetes, or chronic inflammation.

## 8. Vitamin D as a Biomarker in Cardiovascular Medicine

Beyond its potential causal involvement in cardiovascular pathophysiology, vitamin D functions as a clinically informative biomarker that reflects cumulative environmental exposure, metabolic health, and overall physiological reserve [318,319,320,321,322,323,324,325,326,327,328,329,330,331,332,333]. Among available measures, serum 25-hydroxyvitamin D [25(OH)D] is the preferred indicator of vitamin D status because of its relatively long circulating half-life and biochemical stability. In contrast, the hormonally active metabolite 1,25-dihydroxyvitamin D is tightly regulated by calcium, phosphate, and parathyroid hormone and may remain within the normal range even in states of deficiency, rendering it a less reliable marker of systemic vitamin D availability or tissue-level activity [334]. Circulating 25(OH)D concentrations provide an integrated assessment of both endogenous cutaneous synthesis and dietary intake, capturing the influence of multiple determinants relevant to cardiovascular risk. These include sun exposure, seasonal variation, adiposity and fat sequestration, systemic inflammation, hepatic function, and renal clearance. As such, low 25(OH)D levels often co-occur with adverse cardiometabolic profiles and chronic disease states rather than representing an isolated nutritional deficiency.

Importantly, thresholds defining vitamin D deficiency, insufficiency, and sufficiency remain controversial and may differ depending on the clinical outcome of interest. Epidemiological studies suggest a nonlinear association between 25(OH)D levels and cardiovascular risk, characterized by a steep increase in adverse events at low concentrations and a plateau in risk reduction once moderate sufficiency is achieved [335]. Consistent with this pattern, extremely high circulating 25(OH)D levels have not demonstrated additional cardiovascular benefit. In some observational studies, a U-shaped relationship has been reported, in which both low and very high concentrations are associated with increased risk, underscoring the importance of maintaining physiological rather than supraphysiological vitamin D levels [336].

Recent attention has focused on free or bioavailable vitamin D, which represents the fraction of circulating 25(OH)D not bound to vitamin D–binding protein or albumin and therefore more readily available to target tissues. Genetic polymorphisms affecting vitamin D–binding protein, as well as disease states that alter protein synthesis or loss, can substantially influence total circulating 25(OH)D without necessarily affecting the biologically active fraction [337]. This distinction is particularly relevant in conditions such as chronic inflammation, liver disease, nephrotic syndrome, and in populations of African ancestry, where total 25(OH)D concentrations may underestimate true biological sufficiency [338].

Clinically, low serum 25(OH)D levels consistently predict adverse cardiovascular outcomes, including incident cardiovascular disease and mortality, even after adjustment for traditional risk factors, supporting its role as a prognostic biomarker [339]. However, evidence from supplementation trials suggests that vitamin D status may predominantly reflect underlying disease susceptibility and overall health status rather than represent a universally effective, directly modifiable therapeutic target.

Vitamin D status, as measured by serum 25-hydroxyvitamin D [25(OH)D], might serve more effectively as a “biomarker of favorable lifestyle exposure” rather than an independent mediator of cardiovascular benefit [340,341,342,343,344,345]. While physical activity, weight reduction, and sensible sun exposure robustly increase 25(OH)D levels and powerfully reduce cardiovascular disease risk through direct mechanisms—such as improving endothelial function, lowering blood pressure, reducing systemic inflammation, and ameliorating metabolic profiles—the vitamin D elevation itself could be a secondary correlate of these behaviors, as suggested by the fundamental disconnect between observational studies and randomized trials: although low 25(OH)D is consistently associated with higher cardiovascular risk, direct vitamin D supplementation fails to replicate the significant cardiovascular benefits of the lifestyle interventions that raise it. Vitamin D supplementation remains essential for correcting deficiency in at-risk individuals, primarily for musculoskeletal health, but does not seem to be a stand-alone strategy for cardiovascular prevention.

## 9. Special Populations

Certain populations are particularly vulnerable to vitamin D deficiency and its cardiovascular consequences. Older adults experience reduced cutaneous synthesis, dietary intake, and renal activation of vitamin D. Low 25(OH)D in the elderly has been linked to frailty, sarcopenia, impaired functional capacity, and elevated cardiovascular risk [346,347,348,349,350,351,352,353,354,355]. While supplementation improves musculoskeletal outcomes in deficient older adults, cardiovascular benefits remain inconsistent, likely due to late intervention in established disease processes. Chronic kidney disease alters vitamin D metabolism via impaired 1-alpha-hydroxylation, promoting secondary hyperparathyroidism, vascular calcification, and left ventricular hypertrophy [356]. Observational studies associate low 25(OH)D with increased cardiovascular mortality, yet interventional trials with nutritional vitamin D and active analogs show mixed effects [357], reflecting the complex interplay of mineral metabolism, uremia, and vascular pathology. Patients with diabetes and metabolic syndrome frequently exhibit deficiency, influenced by adiposity, inflammation, and lifestyle factors. Vitamin D modulates insulin secretion, sensitivity, and systemic inflammation, linking it mechanistically to cardiometabolic risk [358]. However, supplementation trials rarely show meaningful improvements in glycemic control or cardiovascular endpoints, indicating that deficiency may serve as a marker rather than a direct driver of disease. Heart failure, particularly heart failure with preserved ejection fraction, is associated with high prevalence of deficiency; low 25(OH)D correlates with disease severity, exercise intolerance, and increased morbidity [359]. While supplementation may improve surrogate endpoints, definitive evidence of benefit for hard clinical outcomes is lacking. Ethnic and racial disparities complicate interpretation. Lower total 25(OH)D in certain populations does not consistently reflect reduced bioavailability, yet these populations often exhibit higher cardiovascular risk [360]. Accurate risk assessment requires consideration of genetic, environmental, and social determinants in conjunction with measured vitamin D levels.

## 10. Safety, Toxicity, and Drug Interactions

Vitamin D is widely recognized as safe when administered at physiologic doses; however, supraphysiologic intake can produce clinically significant toxicity [361,362]. Safe and optimal vitamin D levels remain an area of active investigation, particularly in cardiovascular and cardiometabolic medicine. Serum 25-hydroxyvitamin D is the accepted marker of vitamin D status due to its stability and reflection of total body stores. Most expert panels consider concentrations below 20 ng/mL (50 nmol/L) to indicate deficiency and levels between 20 and 30 ng/mL to reflect insufficiency, while concentrations in the range of approximately 30 to 50 ng/mL are generally viewed as sufficient for overall health. Observational studies consistently demonstrate that cardiovascular risk rises steeply at lower 25-hydroxyvitamin D concentrations, with diminishing benefit beyond moderate sufficiency. Importantly, levels above 50 to 60 ng/mL have not been associated with additional cardiovascular benefit and, in some cohorts, have shown neutral or even adverse associations, supporting a nonlinear or U-shaped relationship between vitamin D status and clinical outcomes. From a safety perspective, sustained concentrations above 100 ng/mL increase the risk of hypercalcemia, nephrolithiasis, and vascular calcification and are therefore not recommended. Current evidence supports maintaining vitamin D levels within a physiological range that avoids deficiency while also preventing excessive supplementation, emphasizing individualized assessment based on comorbidities, baseline status, and overall risk profile rather than universal high-dose replacement strategies [317,363,364,365].

Understanding the safety profile is essential in cardiovascular medicine, as patients frequently have comorbidities, polypharmacy, or pre-existing organ dysfunction that may modulate the risk-benefit balance of supplementation. The mechanisms, manifestations, and clinical considerations are discussed below.

### 10.1. Toxicity and Hypercalcemia

The principal adverse effect of vitamin D excess is hypercalcemia, resulting from increased intestinal calcium absorption, enhanced bone resorption, and renal calcium retention. Sustained hypercalcemia can produce neuropsychiatric symptoms, gastrointestinal disturbances, polyuria, polydipsia, and, in severe cases, renal failure [366,367,368]. In cardiovascular populations, hypercalcemia may exacerbate pre-existing arrhythmias, particularly in patients with prolonged QT intervals or conduction abnormalities, by increasing intracellular calcium and altering myocardial action potentials [369,370]. Chronic hypercalcemia also accelerates vascular calcification, particularly in the aorta and coronary arteries, contributing to increased arterial stiffness and systolic hypertension, which are independent predictors of cardiovascular morbidity and mortality [371,372,373,374]. Hypercalciuria, a common laboratory manifestation of excessive vitamin D, can precipitate nephrolithiasis and renal tubular injury, further complicating cardiovascular management, especially in patients with pre-existing chronic kidney disease (CKD) [375,376,377,378,379].

### 10.2. Cardiovascular-Specific Considerations

While vitamin D toxicity is uncommon at standard supplementation doses, cardiovascular-specific risks warrant attention. Excess calcium loading can promote arrhythmogenicity by increasing cardiomyocyte intracellular calcium and prolonging action potential duration, potentially triggering supraventricular or ventricular arrhythmias. Experimental studies demonstrate that high-dose vitamin D in VDR-null or genetically susceptible models induces myocardial calcification, fibrosis, and impaired contractility, underscoring that supraphysiologic levels may have direct deleterious effects on cardiac structure and function [53]. In heart failure, excessive calcium influx may exacerbate diastolic dysfunction, arrhythmia susceptibility, and myocardial oxygen demand, highlighting the need for individualized dosing guided by baseline 25(OH)D concentrations and cardiac status [380].

### 10.3. Influence of Renal Function

Renal impairment significantly alters vitamin D metabolism, as the kidney is the primary site of 1α-hydroxylation converting 25(OH)D to active 1,25(OH)_2_D (Figure 1). In CKD, reduced 1α-hydroxylase activity, combined with impaired phosphate and calcium handling, increases the risk of hypercalcemia, hyperphosphatemia, and vascular calcification when vitamin D is supplemented aggressively. Therefore, in patients with CKD, particularly those on dialysis, dosing should prioritize calcifediol or active vitamin D analogs with close monitoring of serum calcium, phosphate, parathyroid hormone (PTH), and 25(OH)D levels to avoid iatrogenic mineral imbalance [381]. Moderate correction of deficiency in CKD improves endothelial function and reduces inflammatory markers without significantly increasing hypercalcemia risk, highlighting a narrow therapeutic window [382].

### 10.4. Drug Interactions

Vitamin D metabolism is mediated by cytochrome P450 enzymes, particularly CYP3A4, rendering it susceptible to pharmacokinetic interactions. Statins, calcium channel blockers, macrolide antibiotics, and certain immunosuppressants may alter 25(OH)D concentrations by modulating CYP3A4 activity [383]. Conversely, vitamin D may influence the metabolism of other drugs that share CYP3A4 pathways, though clinically significant interactions are rare at standard doses. Thiazide diuretics, commonly used in hypertension management, can increase serum calcium by reducing renal excretion, potentiating the risk of hypercalcemia in patients concurrently receiving vitamin D supplementation [384]. Glucocorticoids impair 1α-hydroxylase activity and VDR-mediated signaling, reducing both endogenous activation and responsiveness to supplementation, which may necessitate higher dosing or alternative formulations to achieve target serum 25(OH)D levels [385]. Loop diuretics, ACE inhibitors, and angiotensin receptor blockers generally have minimal impact on vitamin D pharmacokinetics but may influence calcium and phosphate balance, requiring integrated monitoring.

### 10.5. Formulation and Pharmacokinetics

The formulation of vitamin D significantly influences its pharmacokinetic profile and safety. Cholecalciferol (vitamin D3) is absorbed slowly and requires hepatic 25-hydroxylation to produce 25(OH)D, achieving peak serum concentrations over days to weeks. Calcifediol (25-hydroxyvitamin D3) bypasses hepatic hydroxylation, producing more rapid and stable serum levels, which is advantageous in malabsorptive states, obesity, or CKD [386]. Ergocalciferol (vitamin D2) has a shorter half-life and lower potency in raising serum 25(OH)D, potentially requiring more frequent dosing. The route of administration—oral versus intramuscular—also impacts bioavailability, with intramuscular injections producing prolonged serum exposure but higher peak concentrations, which may transiently increase calcium absorption and require monitoring [387,388,389].

High-dose intermittent regimens, such as monthly or quarterly bolus doses, produce transient supraphysiologic peaks that differ from steady-state daily supplementation, potentially altering VDR activation, gene transcription, and downstream cardiovascular effects. Excessive bolus dosing may paradoxically increase fall or fracture risk in older adults, possibly via calcium overload or dysregulated muscle function, and could theoretically modulate vascular calcification risk, although robust cardiovascular outcome data are limited [390,391]. Daily or weekly physiologic dosing maintains more stable 25(OH)D concentrations, which may better mimic endogenous vitamin D action and reduce adverse events. Of note, new formulations for the administration of vitamin D are currently under investigation [392,393,394,395,396].

### 10.6. Monitoring and Risk Mitigation

To maximize safety and minimize toxicity, supplementation should be individualized based on baseline serum 25(OH)D levels, renal function, comorbidities, and concomitant medications. Routine monitoring of calcium, phosphate, creatinine, and 25(OH)D is recommended in high-risk populations, particularly CKD, heart failure, or patients receiving high-dose therapy. In these groups, incremental titration is safer than empiric high-dose administration. Patients on thiazides or other agents that increase calcium levels require closer surveillance, and glucocorticoid users may require adjusted doses to achieve target serum 25(OH)D without inducing hypercalcemia. Integration of formulation, dose, and frequency with patient-specific factors is essential to optimize efficacy while maintaining safety.

Older adults, patients with obesity, malabsorption, or liver disease, and those with genetic variations in VDR or vitamin D–binding protein may have altered pharmacokinetics or sensitivity to supplementation. In these populations, careful titration, selection of formulation (e.g., calcifediol for rapid correction), and monitoring are particularly important to prevent toxicity. Cardiovascular comorbidities such as heart failure, arrhythmias, or CKD further influence the risk-benefit profile, emphasizing that vitamin D therapy should be individualized and closely monitored in clinical practice [397].

Overall, Vitamin D is generally safe at physiologic doses, but supraphysiologic or high intermittent dosing carries risks of hypercalcemia, hypercalciuria, nephrolithiasis, vascular and myocardial calcification, and arrhythmia. Cardiovascular patients require individualized consideration of baseline 25(OH)D, renal function, comorbid conditions, concomitant medications, and formulation-specific pharmacokinetics. Drug interactions primarily involve CYP3A4 substrates and inducers, thiazide diuretics, and glucocorticoids. Daily or weekly dosing with careful monitoring is preferred over high-dose bolus regimens to minimize peaks in serum calcium and optimize safety. Clinicians must integrate pharmacologic, patient-specific, and cardiovascular factors to safely implement vitamin D supplementation in clinical practice.

## 11. Precision and Personalized Vitamin D Therapy

Emerging evidence supports precision approaches, integrating baseline deficiency, genetics, comorbidities, and timing. Individuals with severe deficiency, elevated renin activity, or early-stage endothelial dysfunction may derive the most cardiovascular benefit, while replete individuals show limited response [398]. Genetic profiling, including VDR, CYP27B1, and vitamin D–binding protein polymorphisms, may identify responders versus non-responders, guiding targeted supplementation [399]. Vitamin D may act synergistically with lifestyle interventions, antihypertensive therapy, or anti-inflammatory strategies, particularly in obese or diabetic populations [400]. Mechanistic biomarkers, including renin–angiotensin activity, inflammatory cytokines, endothelial function, and vascular calcification indices, may help define individualized targets. Adaptive trial designs incorporating these factors could clarify subgroup-specific benefits, advancing precision cardiovascular therapy.

## 12. Future Directions and Research Gaps

Despite extensive research, key questions regarding vitamin D in cardiovascular medicine remain unanswered. Identification of populations most likely to benefit from supplementation is a priority. Current trials often enroll heterogeneous cohorts, including vitamin D–replete individuals, potentially obscuring effects in those who are truly deficient. Stratification by baseline 25-hydroxyvitamin D concentration, genetic polymorphisms affecting metabolism and receptor function, and mechanistic biomarkers such as renin activity or endothelial function could enable targeted interventions. Optimal dosing regimens require clarification. Daily, weekly, and monthly supplementation differ in pharmacokinetics and receptor engagement, with intermittent high-dose protocols producing transient serum peaks that may alter gene expression and downstream cardiovascular effects. Head-to-head comparisons of formulation, frequency, and route, especially in patients with impaired absorption or renal impairment, are needed to define biologically and clinically effective strategies. Timing of intervention is another critical determinant. Cardiovascular pathology develops over decades, and late supplementation may fail to reverse established structural changes. Early intervention, possibly in midlife or during the onset of subclinical disease, may offer the greatest opportunity for meaningful impact. Longitudinal cohort studies integrating serial vitamin D measurements, imaging, and functional phenotyping could inform preventive strategies. Integration of vitamin D with multi-modal interventions represents a promising avenue. Its pleiotropic effects on inflammation, oxidative stress, and neurohormonal pathways suggest potential synergy with antihypertensive therapy, metabolic management, or lifestyle modification. Adaptive trial designs and factorial interventions may clarify whether vitamin D functions as an enhancer of conventional therapies rather than as a standalone treatment. Mechanistic research remains essential. Beyond RAAS modulation, inflammation, and endothelial effects, vitamin D may influence mitochondrial dynamics, epigenetic regulation, and paracrine signaling within cardiovascular tissues. Advanced omics and imaging approaches can elucidate tissue-specific actions, inform personalized therapy, and identify early mechanistic endpoints that predict clinical benefit.

## 13. Conclusions

Vitamin D is a pleiotropic regulator of cardiovascular physiology and disease. Mechanistic studies demonstrate modulation of cardiomyocyte calcium handling, endothelial function, vascular smooth muscle proliferation, inflammation, oxidative stress, and renin–angiotensin–aldosterone system activity, all of which influence cardiovascular homeostasis and pathology. Observational studies consistently associate low 25-hydroxyvitamin D concentrations with hypertension, atherosclerosis, heart failure, arrhythmias, stroke, and mortality, highlighting its prognostic significance. Randomized trials have largely failed to demonstrate reductions in major cardiovascular events, reflecting heterogeneity in baseline status, dosing, timing, genetics, and comorbidities. These findings indicate that vitamin D may serve primarily as a biomarker of cardiometabolic health rather than a uniformly effective intervention. Deficiency appears to exacerbate vulnerability, whereas supplementation alone is insufficient to overcome strong causal drivers such as obesity, diabetes, or systemic inflammation.

Vitamin D illustrates the limitations of nutrient-focused cardiovascular therapies, as low levels are consistently associated with higher cardiovascular risk, yet randomized trials of supplementation have not demonstrated clear reductions in events. Clinicians should use vitamin D primarily as a tool for risk stratification, identifying patients who may be at higher risk due to deficiency or overall health status, and should correct severe deficiency to support bone health and general well-being. They should not expect that normalizing vitamin D alone will prevent heart attacks, stroke, or replace established cardiovascular therapies, and routine supplementation in patients without deficiency offers no proven cardiovascular benefit. Uncertainties remain regarding whether specific subgroups might derive cardiovascular protection from supplementation, the optimal thresholds for risk stratification, and potential interactions with other nutrients or interventions, highlighting the need to integrate vitamin D assessment into broader, evidence-based cardiovascular prevention strategies rather than treat it as a primary therapeutic target.

Future research should focus on precision strategies that integrate baseline deficiency, genetic profiling, and mechanistic biomarkers. Trials targeting individuals most likely to respond, combined with early intervention and integration with multi-modal therapy, hold the greatest promise for translating mechanistic insights into meaningful cardiovascular benefit. Until such evidence is available, supplementation should primarily aim to correct deficiency for skeletal and metabolic health, while acknowledging potential but unproven cardiovascular advantages.

## Figures and Tables

**Table 1:** Vitamin D Nomenclature.

Term	Chemical Name and Composition	Primary Function	Clinical Relevance
**VITAMIN D1**	Mixture of ergocalciferol and lumisterol (1:1)	Historical compound initially identified as “vitamin D”	No current clinical use; obsolete classification
**VITAMIN D2**	Ergocalciferol (derived from ergosterol, plant/fungal source)	Converted in liver to 25(OH)D2 and subsequently to active metabolites	Less potent than D3 in raising and sustaining serum 25(OH)D; still used in some prescription supplements
**VITAMIN D3**	Cholecalciferol (derived from 7-dehydrocholesterol in skin via UVB exposure; also dietary)	Primary physiologic precursor of vitamin D metabolism in humans	Preferred form in supplementation; more effective than D2 for improving vitamin D status
**VITAMIN D4**	22-Dihydroergocalciferol	Minor sterol-derived vitamin D analog	No established clinical role; mainly of biochemical interest
**VITAMIN D5**	Sitocalciferol (derived from 7-dehydrositosterol)	Experimental vitamin D analog	Limited human data; investigated mainly in preclinical studies
**25(OH)D**	25-Hydroxyvitamin D (calcidiol)	Major circulating storage form of vitamin D	Gold-standard biomarker of vitamin D status; low levels associated with cardiometabolic and cardiovascular risk
**1,25(OH)_2_D**	1,25-Dihydroxyvitamin D (calcitriol)	Active hormonal form binding the VDR	Mediates genomic and non-genomic effects in cardiovascular, renal, immune, and skeletal systems
**24,25(OH)_2_D**	24,25-Dihydroxyvitamin D	Inactive metabolite produced by CYP24A1	Marker of vitamin D catabolism; used to assess vitamin D metabolic balance
**VDR**	Vitamin D Receptor (nuclear receptor)	Transcriptional mediator of 1,25(OH)_2_D signaling	Expressed in cardiomyocytes, vascular cells, and immune cells; involved in inflammation, fibrosis, and remodeling
**DBP**	Vitamin D–Binding Protein	Plasma transport protein for vitamin D metabolites	Determines circulating bioavailable vitamin D; genetic variants influence individual responses
**BIOAVAILABLE VITAMIN D**	Free + albumin-bound 25(OH)D (not DBP-bound)	Biologically accessible fraction of circulating vitamin D	May better reflect tissue-level activity than total 25(OH)D in some populations
**VITAMIN D STATUS CATEGORIES**	Serum 25(OH)D thresholds	Clinical classification of vitamin D sufficiency	Sufficient > 30 ng/mL (>75 nmol/L); Insufficient 20–30 ng/mL (50–75 nmol/L); Deficient < 20 ng/mL (<50 nmol/L); thresholds impact trial interpretation

**Table 2:** Vitamin D Biology.

Component	Description	Key Molecules	Cardiovascular Implications
**SOURCES**	Vitamin D can be obtained from diet, supplements, and skin synthesis	Vitamin D2 (ergocalciferol), Vitamin D3 (cholecalciferol)	D3 is synthesized in the skin via UVB exposure; dietary intake often inadequate to reach sufficiency alone
**SKIN SYNTHESIS**	Conversion of 7-dehydrocholesterol to vitamin D3 upon UVB exposure	7-dehydrocholesterol, UVB	Influenced by latitude, season, skin pigmentation, age; deficiency linked to higher CVD risk in observational studies
**HEPATIC 25-HYDROXYLATION**	First hydroxylation step forming the main circulating form, 25(OH)D	CYP2R1, CYP27A1	Serum 25(OH)D is used clinically to assess vitamin D status; low levels associate with hypertension, endothelial dysfunction
**CIRCULATING TRANSPORT**	Vitamin D metabolites circulate bound to carrier proteins	Vitamin D–binding protein (DBP), albumin	DBP polymorphisms can alter bioavailable vitamin D and may modulate CVD risk
**RENAL 1α-HYDROXYLATION**	Formation of the active hormone, 1,25-dihydroxyvitamin D [1,25(OH)_2_D]	CYP27B1	1,25(OH)_2_D regulates calcium-phosphate homeostasis, vascular smooth muscle function, and RAAS activity
**CATABOLISM -INACTIVATION**	Conversion to inactive metabolites for clearance	CYP24A1	Dysregulation can lead to excess or deficiency; genetic variations may influence cardiovascular outcomes.
**TARGET RECEPTOR BINDING**	Genomic and non-genomic effects via the vitamin D receptor (VDR)	VDR (nuclear receptor)	VDR expressed in endothelial cells, cardiomyocytes, vascular smooth muscle; mediates transcription of genes affecting inflammation, fibrosis, and cardiac remodeling.
**NON-GENOMIC ACTIONS**	Rapid signaling pathways independent of gene transcription	Membrane VDR, caveolin-1, PLC/PKC pathways	Modulates calcium handling, vascular tone, and cardiomyocyte contractility; contributes to cardiovascular homeostasis.
**SYSTEMIC EFFECTS**	Regulation of calcium-phosphate metabolism, immune modulation, RAAS suppression	PTH, renin, cytokines	Vitamin D deficiency may contribute to hypertension, endothelial dysfunction, atherosclerosis, and heart failure risk.

Abbreviations: 25(OH)D, 25-hydroxyvitamin D; 1,25(OH)_2_D, 1,25-dihydroxyvitamin D; CYP, cytochrome P450 enzyme; RAAS, renin–angiotensin–aldosterone system; VDR, vitamin D receptor; DBP, vitamin D–binding protein; PLC/PKC, phospholipase C/protein kinase C.

**Table 3:** Major Randomized Controlled Trials testing how Cardiovascular Outcomes are affected by Vitamin D Supplementation.

Trial	Intervention	Primary CVD Outcome(s)	Key Results	Ref.
**VITAMIN D AND OMEGA-3 TRIAL (VITAL) (GENERAL ADULTS, PRIMARY PREVENTION)**	Vitamin D3 2000 IU daily vs. placebo	Composite MI, stroke, CVD death	No significant reduction: HR 0.97 (95% CI: 0.85–1.12), *p* = 0.69	[9]
**VIDA STUDY (GENERAL ADULTS, PRIMARY PREVENTION)**	Monthly high-dose vitamin D3 (200,000 IU loading then 100,000 IU monthly) vs. placebo	Major cardiovascular events	No significant effect: HR 1.02 (95% CI: 0.87–1.20)	[11]
**D-HEALTH TRIAL (OLDER ADULTS, PRIMARY PREVENTION)**	Monthly vitamin D3 60,000 IU vs. placebo	Major cardiovascular events	HR 0.91 (95% CI: 0.81–1.01); MI HR 0.81 (95% CI: 0.67–0.98) but overall not statistically conclusive	[14]
**FIND (FINNISH OLDER ADULTS)**	Daily vitamin D3 1600 IU or 3200 IU vs. placebo	Major CVD events	No significant reduction (HRs ~0.97–0.84 NS)	[13]
**EVITA (HEART FAILURE) (ADVANCED HF WITH LOW 25(OH)D)**	Vitamin D3 4000 IU daily vs. placebo	All-cause mortality and cardiovascular complications	No significant difference: HR 1.09 (95% CI: 0.69–1.71)	[12]

Abbreviations: MI, myocardial infarction; CVD, cardiovascular disease; HF, heart failure; HR, hazard ratio; NS, not significant.

**Table 4:** Summary of Major Meta-Analyses of Vitamin D Supplementation Trials with Cardiovascular Endpoints.

Meta-Analysis	Number of Rcts Included	Primary Cardiovascular Findings	Key Effect Sizes	Ref.
**META-ANALYSIS OF RCTS FOR MACE**	5 RCTs	No significant reduction in major adverse cardiovascular events (MACE)	HR ~0.96 for MACE (*p* = 0.77); MI HR ~0.88 (*p* = 0.061, borderline); no stroke or CVD death benefit	[312]
**UPDATED CVD OUTCOMES META-ANALYSIS**	>10 RCTs with cardiovascular endpoints	No significant effect on MI, stroke, or cardiovascular death	RR ~0.99 for MACE; no significant differences in MI, stroke, HF, or CVD death	[8]
**LARGE SYSTEMATIC REVIEW (80 RCTS)**	80 RCTs with >160,000 participants	Lower all-cause mortality; no significant CVD outcome reduction	OR 0.95 for all-cause mortality (*p* = 0.013); no significant CVD morbidity/mortality reduction	[313]
**SYSTEMATIC REVIEW OF CVD MORTALITY AND MI/STROKE**	9–14 RCTs	No reduction in CVD mortality or MI/stroke	RR ~0.96–1.05 across endpoints	[314]

## Data Availability

No new data were created or analyzed in this study. Data sharing is not applicable.
